# Constructing a new health education model for patients with chronic hepatitis B

**DOI:** 10.1097/MD.0000000000023687

**Published:** 2020-12-11

**Authors:** Qinhua Chen, Minyu Jiang, Meixing Zeng, Zhu Liduzi Jiesisibieke, Pei-En Chen, Ching-Wen Chien, Tao-Hsin Tung

**Affiliations:** a Department of Nursing, Maoming People's Hospital, Maoming, Guangdong; b Institute for Hospital Management, Tsinghua University, Shenzhen Canpus, Shenzhen, Guangdong, China; c Institute of Health Policy and Management, Collgue of Public Health, National Taiwan University, Taipei, Taiwan, China; d Taiwan Association of Health Industry Management and Development, Taipei, Taiwan, China; e Department of Medical Research and Education, Cheng Hsin General Hospital, Taipei, Taiwan, China.

**Keywords:** chronic hepatitis B patients, health education, innovative health education model, project-achievement type, quality control circle

## Abstract

To explore the effects of the project-achievement quality control circle in constructing a new health education model for patients with chronic hepatitis B.

The quality control circle group was established and the theme of “constructing a new health education model for patients with chronic hepatitis B” was selected. The circle staff determined that this quality control circle was of project-achievement according to the quality control story judgment table, and then carry out activities in strict accordance with the 10 steps of project-achievement quality control circle, evaluate the tangible results and non-tangible results before and after the activity.

After the implementation of the activity, the health education integrity of patients with chronic hepatitis B increased from 74.75 ± 11.00 to 95.00 ± 5.55 points (*P* < .001). The awareness of health education increased from 71.90 ± 13.48 to 95.60 ± 2.84 points (*P* < .001), the satisfaction rate of health education increased from 76.60 ± 8.71 points to 98.00 ± 2.03 points (*P* < .001), and the evaluation rate after health education increased from 10% to 100% (*P* < .001).

The circle members have much more confidence in quality control circle activities, the use of techniques, and the knowledge related to scientific research.

## 1 Introduction

Quality control circle (QCC) is a lean management tool. As part of total quality management, it has become an important medical quality management activity in many hospitals.^[1]^ The achievement of QCC refers to various issues such as new business, new processes, new services or development of new products, breakthroughs in the status quo, and creation of attractive qualities. Some studies indicated the role of QCC in medical improvement and highlighted the success of its application in medical care and hospital management.^[2,3]^ In addition, quality control story (QC story) was mainly applied to perform the problem-solving process of QCC.^[4]^

China is a high incidence area of viral hepatitis, especially hepatitis B virus infection rate is the highest, chronic hepatitis B is the main cause of liver cirrhosis and liver cancer, and health education is an important nursing measure for chronic diseases, and it is also an important method to increase awareness, improve treatment adherence, and improve unhealthy lifestyles.^[5]^ Traditional health education is monotonous and rigid, and nurse resources are scarce, repeated explanations greatly waste labor force. To our knowledge, little literature focused on the un-optimized process of health education model and the application of QCC in the field of patients with chronic hepatitis B. The purpose of this study is to construct a new model for the health education of patients with chronic hepatitis B using the QCC activities.

## 2 Methods

### 2.1 Data description

#### 2.1.1 Participants

By collecting the data of health education integrity, health education awareness, health education satisfaction, and post-health-education evaluation rate of 80 chronic hepatitis B patients in infectious department of our hospital before (February 2018) and after the activity (October 2018). The 40 cases were given traditional health education before activities, and the other 40 cases were given a new model of health education after activities. The data before and after the activity were collected and analyzed.

#### 2.1.2 Inclusion criteria

In this QCC, the inclusion criteria were:

(1) Complying with the diagnostic criteria of hepatitis B in the 8th edition of Internal Medicine of People's Health Publishing House, China; all patients were diagnosed by positive detection of hepatitis B virus pathogens (markers) and hepatitis B virus DNA;
(2) They had signed the informed consent and volunteered to participate in this QCC;
(3) All the procedures were carried out voluntarily.

The enrolled subjects were excluded from psychiatric disorders such as aphasia and depression. The QCC did not confine the education level, as long as the participants were conscious, had a good communication, and was not illiterate.

#### 2.1.3 Exclusion criteria

The exclusion criteria were:

(1) Hepatitis A, C, D, E;
(2) central nervous system infection;
(3) psychological disorders;
(4) active withdrawal.

### 2.2 Data processing

**Figure 1:**
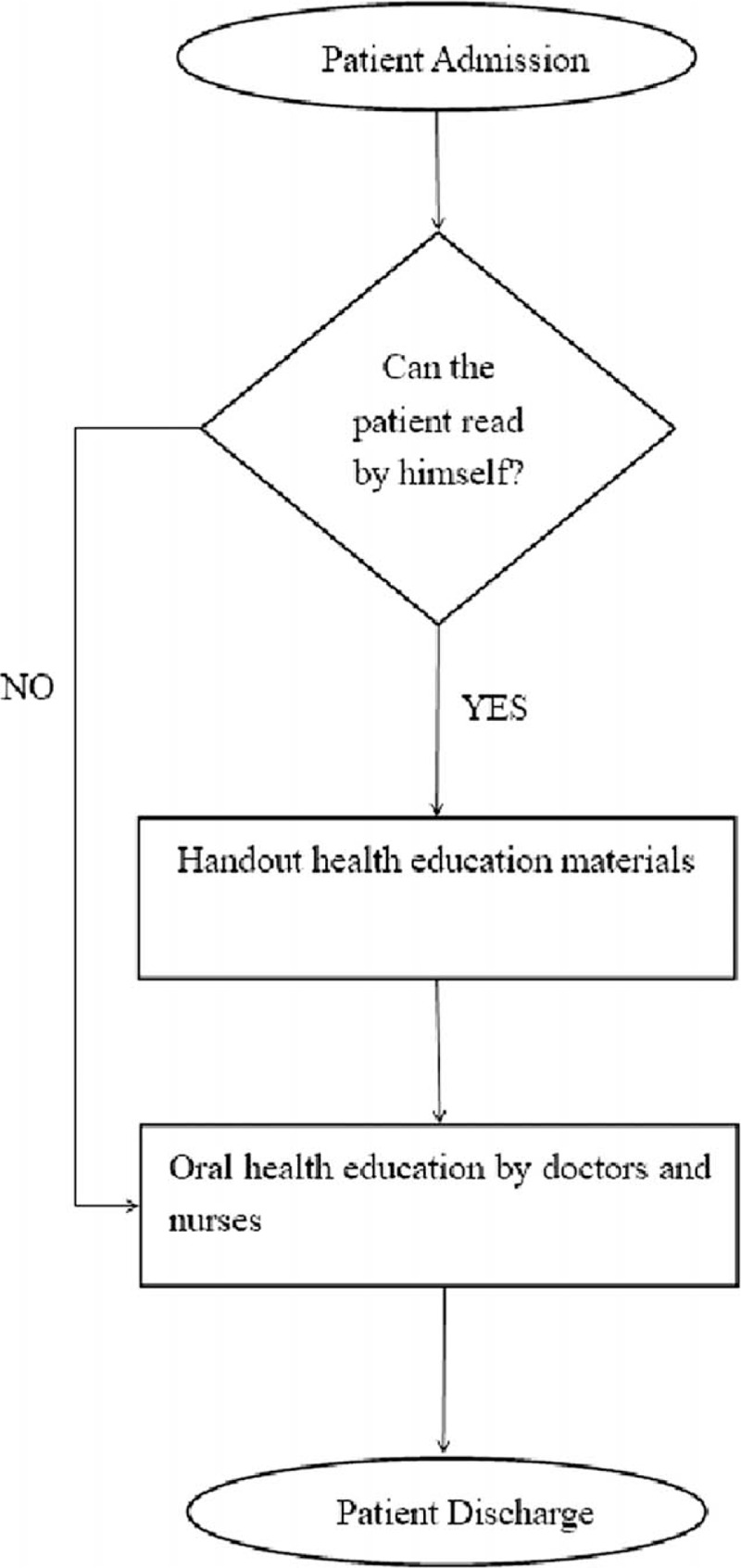
Traditional health education process for chronic hepatitis B patient.

The following steps indicated data progress:

(1) Establishment of quality control circle
  This quality control circle group is an interdisciplinary and inter-departmental cooperation involving the infection department and the hospital propaganda office. It consists of 8 members of the infectious doctor, nurse, and publicity office. The average years they have participated in QCC are 2. Choose 1 as the circle leader and 1 as counselor. Before the activity, all the circle members accepted the training of the project to achieve the QCC, and after the training, the relevant knowledge assessment was carried out, and the members carried out the activities after passing the assessment.
(2) Theme selection and plan setting
  Brainstorming method was used to raise questions, and the superior policies, importance, urgency, and circle ability were scored according to the evaluation method and weight method. Finally, the theme of this activity was “constructing a new health education model for patients with chronic hepatitis B.” According to the QC-STORY decision table, this activity is determined as the topic of quality control circle. Activities follow the Plan-Do-Check-Action procedure, and carry out activities in strict accordance with the 10 steps of theme selection, plan setting, topic clarification, target setting, strategy formulation, optimal policy investigation, optimal policy implementation and review, effect confirmation, standardization and review and improvement.
(3) Topic clarification
  The traditional health education process is sorted out and drawn into a flow chart (Fig. 1). The key link that needs to be broken is the way of health education and the effect evaluation after the patient education.
  Based on the theme, members analyzed from 3 aspects that is personnel, methods and norms: the way each health educator educates and the educational content received by each patient are very different, and the patients have no effect evaluation after receiving health education. In response to the status quo, the circle members evaluated the importance, feasibility, and ability of the circle, and selected 9 major key points (Table 1).
  Through a questionnaire survey of 40 patients with chronic hepatitis B who were admitted to our department in February 2018, the survey was divided into 3 main contents: the completeness, recognition, and satisfaction of health education for patients with chronic hepatitis B. The evaluation is based on 7 dimensions, that is, psychological guidance, rest guidance, diet guidance, inspection guidance, examination guidance, drug guidance, and protective guidance. The integrity level is divided into 2 dimensions; the recognition level is divided into 3 dimensions included clear, partially clear, and unclear; Satisfaction rate is divided into 3 dimensions that is satisfied, generally satisfied, and dissatisfied. The survey data showed that the patient's integrity of traditional health education was 74.75 ± 11.00 points, awareness was 71.90 ± 13.48 points, and satisfaction was 76.60 ± 8.71 points.
(4) Target setting
  According to the current status, the team members consult the literature, experts, check the benchmark, and set the target value of the activity according to the formula: the completeness of the health education of the chronic hepatitis B patients is 95 points, the recognition level is 95 points, 95 points of satisfaction level, and an evaluation rate of 100%.

**Table 1:** Selection of key points.

Items	Constructing a new health education model for patients with chronic hepatitis B
Personnel		
Key Point 1	Improve health education
Key Point 2	Establish a health education program that meets the needs of patients
Key Point 3	Revise the new health education process
Key Point 4	Establish a new health education system
Methods		
Key Point 5	WeChat, public health education platform
Key Point 6	Micro-lecture online health education
Key Point 7	Video health education
Norms		
Key Point 8	Establish a post-evaluation program for health education
Key Point	9 Establish a health education evaluation too

### 2.3 Strategy formulation and optimal implementation

#### 2.3.1 Strategy formulation and optimal policy investigation

The 9 key points would be integrated into 4 major key points. Through the system diagram method, each was refined and expanded in sequence. Through the circle members rating, 14 countermeasures were selected. Designing obstacle removal tracking table to evaluate and integrate 14 countermeasures from 3 aspects of feasibility, economy, and effectiveness. Finally, the 3 major policy groups were selected, that is,

(1) improve the health education system;
(2) establish intelligent health education mode; and
(3) establishes a homogeneous education evaluation system.

#### 2.3.2 Optimal implementation and review

Formulation Group I, to improve the health education system.

(1) Build a health education system. The health education structure will be constructed from the 3 aspects that is decision-making level, implementation level and security level, and established responsibilities of personnel at all levels to ensure the implementation of health education.
(2) Develop the evaluation content of patients after health education. Use the consensus to develop the post-evaluation program of patient health education to ensure that patients receive the same standard effect evaluation.

Formulation Group II, to establish an intelligent health education method.

(1) The patient would be given video teaching after admission without exceptions.^[6]^ The director of the propaganda office jointly designed and filmed a multi-lingual version of the chronic hepatitis B health education video. After admission, patients can choose videos according to languages while waiting for the procedure to ease anxiety during waiting and improve the integrity of patients’ health educational knowledge.
(2) Micro lecture with a precise goal and content comes with better effective giving specific knowledge.^[7]^ Using micro-lecture for health education, since patients have individualized health problems during hospitalization, using micro-lecture for individual health education, to strengthen patients’ awareness of health education.

Establish a WeChat communication platform included:

(1) Establish a public platform for the infection department, arrange personnel to push relevant knowledge every week, and send the link to the patient to read, to ensure the patient's to learn latest disease-related knowledge.
(2) Establish a health education consulting platform for chronic hepatitis B. After discharging from hospital, patients consult on the platform to continue to learn the knowledge. Doctors and nurses in charge of beds answer on the spot to meet the educational needs of patients after discharging from hospital.

Formulation Group III, establish a homogenization education evaluation system.

(1) Using questionnaire to survey included design questionnaires and generate QR codes.
(2) After the patients finish the questionnaires, they can see the results after submission.
(3) After watching the video, the patients were asked to conduct a questionnaire survey. Through consulting experts outside the circle, we determined the standard was 90 points. If less than 90 points, the patients were asked to watch the video again. Health education guided by effect evaluation ensures patients’ understanding of health education.^[8–10]^

### 2.4 Statistical analysis

Statistical analysis was performed using SPSS 22.0 for Windows. A *P*-value of <.05 was considered to represent a statistically significant difference between 2 test populations. The paired *t*-test method was adopted to assess the difference in the mean value of continuous variables between before and after groups. The Radar chart showed the QC story evaluation The Radar chart was used to evaluate the effectiveness of QC story.

## 3 Results

### 3.1 Tangible results

**Figure 2:**
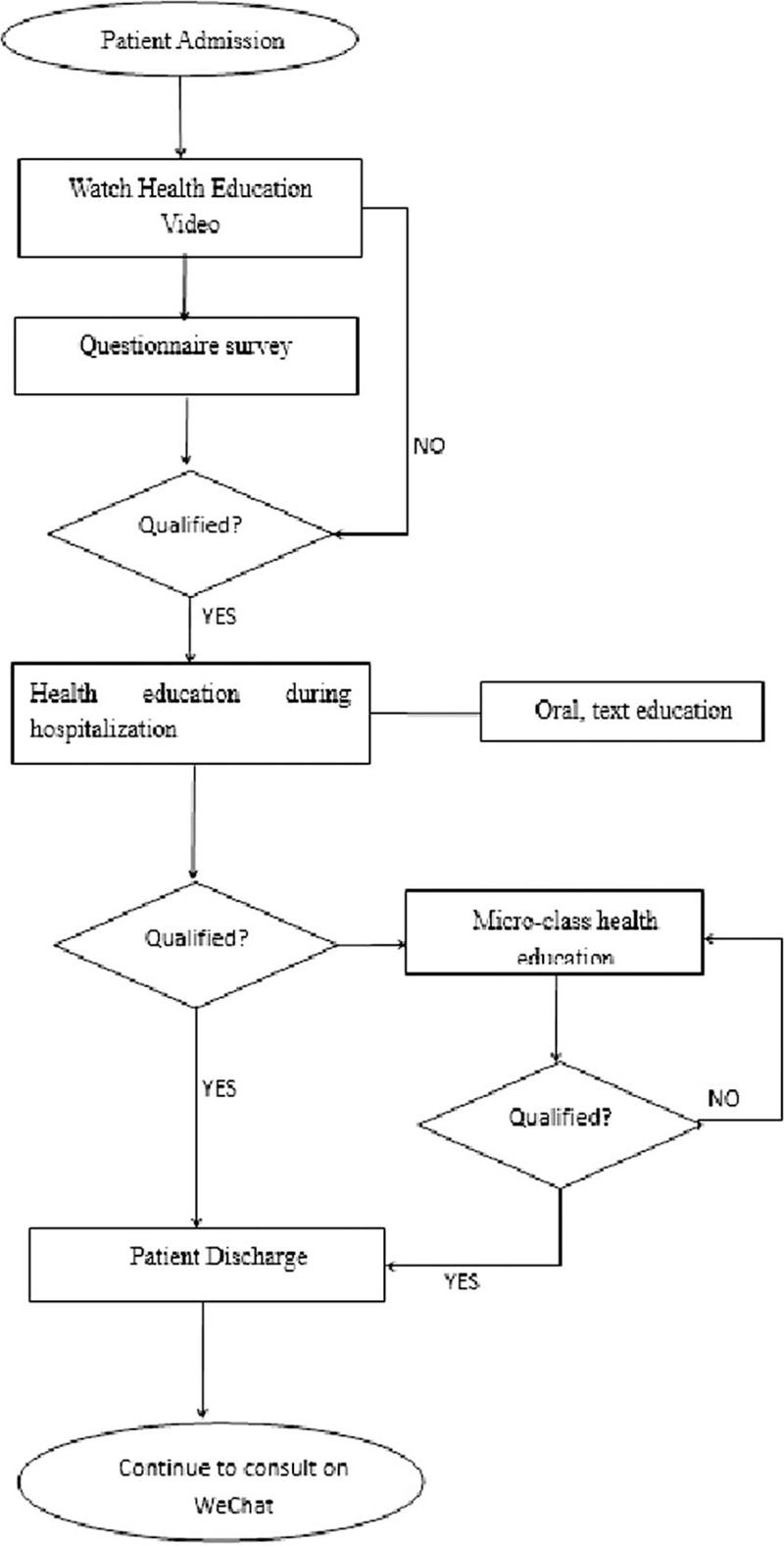
Construction process of new health education model for patients with chronic hepatitis B.

**Figure 3:**
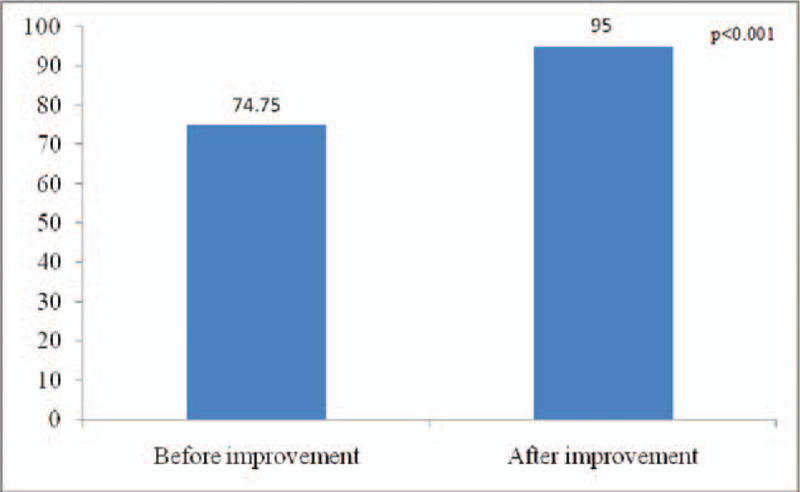
The health education integrity of patients with chronic hepatitis B before and after improvement.

**Figure 4:**
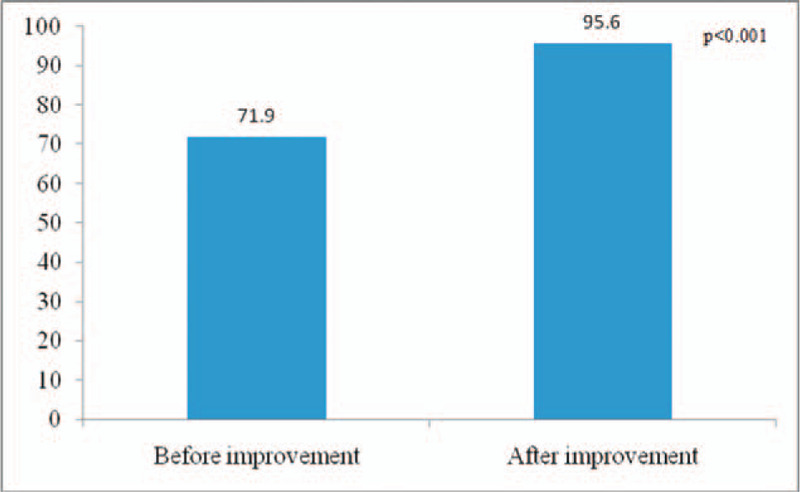
The health education awareness of patients with chronic hepatitis B before and after improvement.

After the improvement, the health education process of the chronic hepatitis B was illustrated in Figure 2. The impact of the new health education model on patients with chronic hepatitis B: the health education integrity of patients with chronic hepatitis B increased from 74.75 ± 11.00 to 95.00 ± 5.55 points (*P* < .001); the awareness of health education for patients with chronic hepatitis B increased from 71.90 ± 13.48 to 95.60 ± 2.84 points (*P* < .001), and the data before and after the process was statistically significant (Figs. 3 and 4).

**Figure 5:**
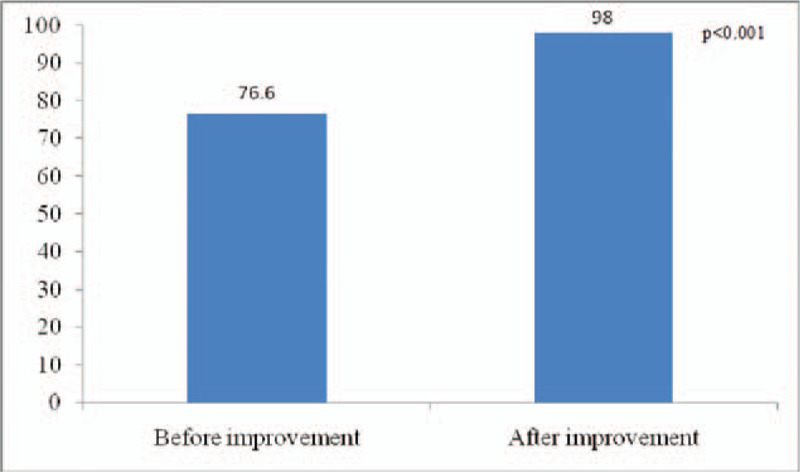
The satisfaction of health education of patients with chronic hepatitis B before and after improvement.

**Figure 6:**
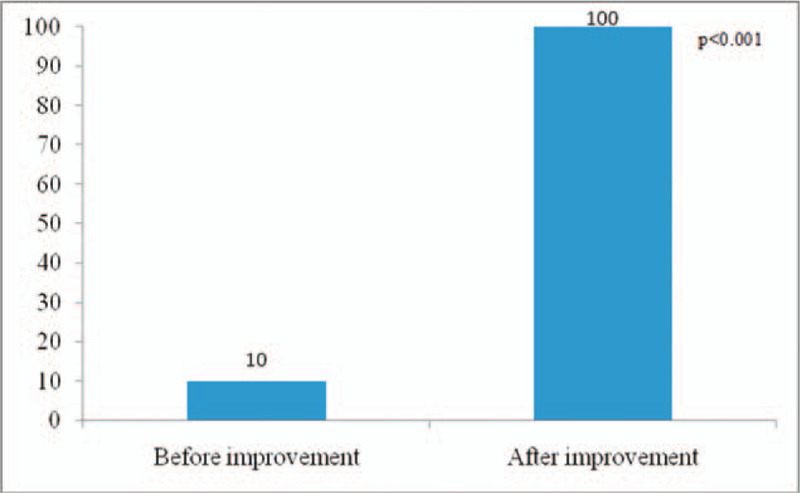
The evaluation rate of health education of patients with chronic hepatitis B before and after improvement.

Health education satisfaction and evaluation rate of patients with chronic hepatitis B: The satisfaction of health education for patients with chronic hepatitis B increased from 76.60 ± 8.71 points to 98.00 ± 2.03 points (*P* < .001); the evaluation rate of health education for patients with chronic hepatitis B increased from 10% to 100% (*P* < .001). The data before and after the process transformation were statistically significant (Figs. 5 and 6). In addition, Table 2 indicates the significant difference of integrity, awareness, and satisfaction of each guidance between before and after improvement.

**Table 2:** The comparisons integrity, awareness, and satisfaction of each guidance between before and after improvement (n = 40).

	Integrity			Awareness			Satisfaction		
Items	Before mean ± SD	After mean ± SD	*P*-value for paired *t*-test	Before mean ± SD	After mean ± SD	*P*-value for paired *t*-test	Before mean ± SD	After mean ± SD	*P*-value for paired *t*-test
Psychological guidance	7.75 ± 4.23	9.50 ± 2.21	.03	8.80 ± 2.07	9.70 ± 1.07	.02	8.80 ± 2.43	9.90 ± 0.63	.01
Rest guidance	7.25 ± 4.52	9.00 ± 3.04	.03	7.60 ± 3.36	9.60 ± 1.22	.001	7.90 ± 2.56	9.60 ± 1.22	.001
Diet guidance	6.75 ± 4.74	9.25 ± 2.67	.006	8.20 ± 2.86	9.50 ± 1.34	.01	8.10 ± 3.00	9.70 ± 1.07	.003
Inspection guidance	7.25 ± 4.52	9.50 ± 2.21	.01	7.90 ± 3.00	9.80 ± 0.88	.001	9.00 ± 2.17	9.80 ± 0.88	.03
Examination guidance	8.00 ± 4.05	9.50 ± 2.21	.03	7.90 ± 2.56	9.80 ± 0.88	<.001	8.50 ± 2.82	9.90 ± 0.63	.01
Drug guidance	14.50 ± 7.83	19.25 ± 2.67	.001	14.60 ± 5.32	19.20 ± 1.62	<.001	16.30 ± 3.77	19.80 ± 0.88	<.001
Protective guidance	21.50 ± 8.71	29.00 ± 3.04	<.001	16.90 ± 5.20	28.00 ± 2.72	<.001	18.00 ± 5.36	29.30 ± 1.54	<.001

### 3.2 Nontangible results

**Figure 7:**
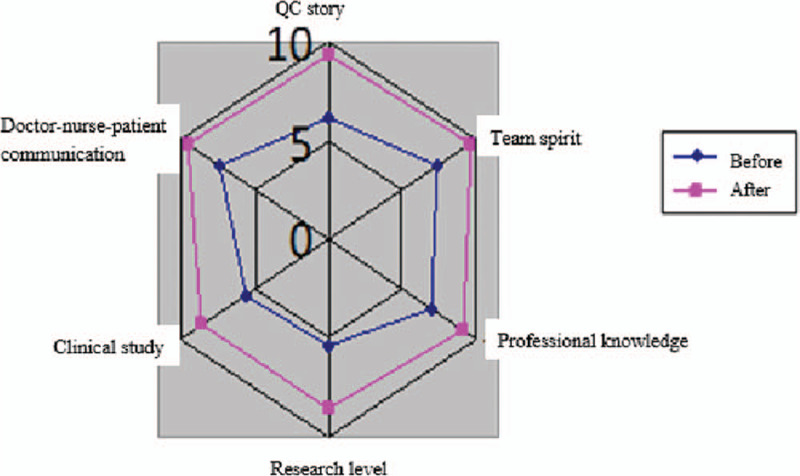
Radar chart.

All circle members was evaluated the using skills of QCC, team spirit, professional knowledge, research level, clinical research, and communication skills of doctors and nurses. Activity growth value equals to average score after activity minus average score before activity, positive for improvement, negative for negative (Table 3 and Fig. 7). The results show that the evaluation programs of the circle members’ skills, professional knowledge, and scientific research level have been significantly improved.

**Table 3:** The comparison of each item between before and after improvement.

	Before Improvement		After Improvement		
Items	Total	Average	Total	Average	Increasing Level
The skills of using QCC	49	6.1	74	9.3	3.2
Team spirit	59	7.4	78	9.7	2.3
Professional knowledge	56	7	73	9.1	2.1
Research level	55	5.4	79	8.6	3.5
Clinical research	47	5.9	77	8.6	3.7
Communication skills of doctors and nurses	60	7.4	77	9.6	2.2

QCC = quality control circle.

## 4 Discussion

### 4.1 The problem-achieving QCC improves the health education effect of patients with chronic hepatitis B

In recent years, the quality control circle activities have been rapidly developed in domestic medical institutions, which greatly promoted the improvement of medical quality. Chronic hepatitis B patients need in-depth health education knowledge guidance, and there are different levels of knowledge needs for health education. The traditional health education model can not meet the health needs of patients, and could not promote the health of patients. First, video health education improves the health education effect of patients with chronic hepatitis B. This time, the quality control circle activities with the theme of “construction of a new health education model for patients with chronic hepatitis B” reformed the traditional 1-way information mainly based on oral and text publicity. Establish a health education mode based on multimedia, internet, film, and television. By giving a video message when the patient is newly admitted to the hospital, the anxiety of the patient can be alleviated; and the video of health education is shot by animation, which is interesting and has multi-lingual choice to meet the needs of different patients. Video playback can be repeated and repeated, to meet the needs of patients to watch while not causing the burden of human resources. Second, the effect of health education of chronic hepatitis B patients can be strengthened by micro-lecture online health education plus effect evaluation-oriented health education. Studies have shown that after routine health education, 40% to 80% of the information is directly forgotten or about 50% of the information is not fully understood. To establish micro-class health education, in view of the doubtful points of patients’ health knowledge, medical staff recorded 5 to 10 minutes of micro-lecture video and sent QR codes to the patients to watch, so that the patients could watch it repeatedly until they understood and followed the rehabilitation guidance. In addition, the questionnaire was used to evaluate effects after the video training of patients with chronic hepatitis B. The patients who did not meet the standard were guided to continue watching the video to ensure that each patient with chronic hepatitis B had relevant health education content. This activity improves the integrity, awareness, satisfaction, and evaluation rate of health education for patients with chronic hepatitis B, and achieves the purpose of this activity.

### 4.2 The QCC has improved the professional level of the circle members

This event has reached a model management circle for the project, which requires strong professional knowledge of the circle members. According to the research of the Guangdong Science and Technology Information Research Institute, there is no relevant literature report in China. The circle members consulted a large number of similar documents and the practice of benchmarking hospitals. In Taiwan, they referenced the advanced health education and evaluation methods. Through continuous learning and exploration, they finally succeeded in constructing a new health education model for patients with chronic hepatitis B, replacing the traditional health education method and the professional level of the circle members have also been greatly improved.

### 4.3 The QCC has improved the level of research and assistance of the circle members

The QCC is a preliminary study of scientific research projects, which makes the clinical nursing work and scientific research more closely integrated, and can promote the output of scientific research results. Through the activity to stimulate the scientific research consciousness of the circle members, through the literature, encourage circle members to participate in scientific research, data collation, and statistical analysis, etc., to improve the scientific research ability of the circle. Second, this activity is cross-departmental assistance, which requires a high degree of tacit understanding and effective communication between circle members. In the course of the activity, circle members’ assistance ability has been greatly improved.

### 4.4 Methodological considerations

Although the success in this QCC, several limitations should also be noted. First, it is conducted in a single center and data collected. A large and multi-center study was definitively needed to verify the efficacy and accuracy of QCC. Second, the generalization and external validity should be further discussed. Thirdly, the results of this study should not be extrapolated to hospitals in other regions of China. Future studies using random sampling of hospitals over a wider range of regions would make the research more discursive.

## 5 Conclusion

The QCC has built a health care model for patients with chronic hepatitis B, and it is a benchmark for other departments. A new health education model for chronic hepatitis B patients constructed by our department after the publication of QCC results in hospitals organized citizens to watch video health education monthly in health education centers. Carried out by oncology, endocrinology, and respiratory departments, it was highly praised by the public and doctors and nurses.

## Author contributions

**Conceptualization:** Pei-En Chen, Ching-Wen Chien, Tao-Hsin Tung.

**Data curation:** Qinhua Chen, Minyu Jiang, Meixing Zeng.

**Methodology:** Tao-Hsin Tung.

**Software:** Qinhua Chen, Minyu Jiang, Meixing Zeng, Zhu Liduzi Jiesisibieke.

**Supervision:** Pei-En Chen, Ching-Wen Chien, Tao-Hsin Tung.

**Writing – original draft:** Qinhua Chen, Minyu Jiang, Meixing Zeng, Zhu Liduzi Jiesisibieke.

**Writing – review & editing:** Pei-En Chen, Ching-Wen Chien, Tao-Hsin Tung.
